# Economic Evaluation of Companion Diagnostic Testing for *EGFR* Mutations and First-Line Targeted Therapy in Advanced Non-Small Cell Lung Cancer Patients in South Korea

**DOI:** 10.1371/journal.pone.0160155

**Published:** 2016-08-02

**Authors:** Eun-A Lim, Haeyoung Lee, Eunmi Bae, Jaeok Lim, Young Kee Shin, Sang-Eun Choi

**Affiliations:** 1 College of Pharmacy, Korea University, Sejong, Korea; 2 College of Pharmacy, Seoul National University, Seoul, Korea; University of South Alabama Mitchell Cancer Institute, UNITED STATES

## Abstract

**Background:**

As targeted therapy becomes increasingly important, diagnostic techniques for identifying targeted biomarkers have also become an emerging issue. The study aims to evaluate the cost-effectiveness of treating patients as guided by epidermal growth factor receptor (*EGFR*) mutation status compared with a no-testing strategy that is the current clinical practice in South Korea.

**Methods:**

A cost-utility analysis was conducted to compare an *EGFR* mutation testing strategy with a no-testing strategy from the Korean healthcare payer’s perspective. The study population consisted of patients with stage 3b and 4 lung adenocarcinoma. A decision tree model was employed to select the appropriate treatment regimen according to the results of *EGFR* mutation testing and a Markov model was constructed to simulate disease progression of advanced non-small cell lung cancer. The length of a Markov cycle was one month, and the time horizon was five years (60 cycles).

**Results:**

In the base case analysis, the testing strategy was a dominant option. Quality-adjusted life-years gained (QALYs) were 0.556 and 0.635, and total costs were $23,952 USD and $23,334 USD in the no-testing and testing strategy respectively. The sensitivity analyses showed overall robust results. The incremental cost-effectiveness ratios (ICERs) increased when the number of patients to be treated with erlotinib increased, due to the high cost of erlotinib.

**Conclusion:**

Treating advanced adenocarcinoma based on *EGFR* mutation status has beneficial effects and saves the cost compared to no testing strategy in South Korea. However, the cost-effectiveness of *EGFR* mutation testing was heavily affected by the cost-effectiveness of the targeted therapy.

## Introduction

Targeted therapy is becoming increasingly emphasized for cancer patients. If a targeted therapy is used to treat patients without the corresponding biomarker, it may result in not only increased burdens of disease, but also unfavorable therapeutic consequences, as targeted therapy is associated with shorter overall survival and progression-free survival (PFS) than standard chemotherapy in treating mutation-negative patients [1].

Therefore, techniques identifying mutation biomarkers are an important issue in the use of targeted therapy. *In vitro* diagnostics (IVD) is a technique that can detect diseases or infection in laboratories using commercialized devices. A laboratory-developed test (LDT) is an IVD that is designed, manufactured, and used within a single laboratory, and follows the protocol of that individual laboratory for testing [2]. Companion diagnostics (CDx) is characterized as an IVD providing information for the safe and effective use of targeted therapy [3]. Accordingly, an assessment of CDx is performed by evaluating diagnostic accuracy and clinical utility, which is defined as the extent of improved clinical outcome of a targeted therapy.

Lung cancer is one of the most common causes of death in the world [4]. Diagnostic techniques for epidermal growth factor receptor (*EGFR*) mutations are important in treatment of advanced non-small cell lung cancer (NSCLC) thanks to the availability of tyrosine-kinase inhibitors (TKIs). *EGFR* mutations in NSCLC are more common in adenocarcinomas, females, never-smokers and ethnic Asians [5]. The prevalence of EGFR mutations is known to be 20–40% in Asian populations and 5–20% in Caucasians [5]. The National Comprehensive Cancer Network (NCCN) Guidelines recommends *EGFR* mutation testing in cases of non-squamous cell carcinoma [6].

Traditionally, platinum-based chemotherapy such as cisplatin or carboplatin has been used as the first-line therapy in stage 3b and 4 NSCLC. The combination of cisplatin plus pemetrexed is more effective in patients with non-squamous histology than cisplatin plus gemcitabine, whereas the latter is more effective in patients with squamous cell histology [6–8]. However, if patients test positive for an *EGFR* mutation, monotherapy with a TKI is recommended [6,7].

In Korea, TKIs tend to be used as a second-line therapy, generally without testing for *EGFR* mutations. This treatment pattern may be due to the high cost of TKIs and additional testing. Unfortunately, this therapeutic practice may lead to a lost opportunity to use TKIs earlier in the treatment of *EGFR* mutation-positive patients. Moreover, although genetic testing technology is being actively developed in South Korea, there is still a lack of awareness regarding companion diagnostics of the approval authorities. Therefore, it is necessary to provide evidence of cost-effectiveness in order to make the best use of the new technology and to diffuse the concept of CDx.

In the past, studies of cost-effectiveness related to this issue were limited; studies now are more likely to be published. Previous studies were conducted with different conditions: gefitinib as a first-line therapy [5, 9], erlotinib as a first-line therapy [10,11], erlotinib as a second-line therapy [12,13], or an evaluation of the cost-effectiveness among test techniques with erlotinib as a first-line therapy [14]. The cost-effectiveness of testing strategies, compared to the no-testing strategy, is still unclear because the results vary across studies due to differences in models, epidemiological data, clinical efficacy data or healthcare costs. For example, the prevalence applied in the studies varied by ethnicity, ranging from 12.8% to 32%. Chemotherapy regimens used were different from one study to another such as cisplatin plus docetaxel [5], carboplatin plus docetaxel [11], carboplatin plus paclitaxel [9] and so forth. Also, a study [5] did consider second-line treatment in the model whereas others considered first-line therapy only.

Our interest was in the cost-effectiveness of TKI treatment and CDx in real-world settings. Thus, we aimed to assess the cost-effectiveness of targeted therapy based on *EGFR* mutation status compared with the current clinical practice in South Korean healthcare after application of all the relevant clinical data. The study is expected to provide important evidence for the treatment of NSCLC patients to healthcare policy makers and genetic assay developers, as well as patients, healthcare providers, and insurers.

## Materials and Methods

### Study Population

The study population consisted of patients with advanced (stage 3b and 4) lung adenocarcinoma, because the prevalence of *EGFR* mutations in adenocarcinoma patients is high, and chemotherapy is indicated as the main therapy in stage 3b or 4 NSCLC. In this model, *EGFR* mutation testing was performed to detect the presence of *EGFR* mutations before using erlotinib as the first-line treatment. Erlotinib of TKIs was our main interest because it was marketed more recently (in 2008) than gefitinib in Korea. Afatinib was not considered because of lack of data for the analysis, which was sold after 2014. Furthermore, any cost-effectiveness study for erlotinib has not included the OPTIMAL trial [15] for the analysis, which investigated clinical outcomes for erlotinib and *EGFR* testing in Asians.

### Strategies Compared

We compared an *EGFR* mutation testing strategy with a no-testing strategy. The *EGFR* mutation testing strategy (‘testing strategy’) was defined as treatment guided by the result of *EGFR* mutation testing in advanced NSCLC patients: erlotinib for *EGFR* mutation-positive patients and conventional chemotherapy for wild type patients. In contrast, a strategy with no *EGFR* mutation testing (‘no-testing strategy’) was defined as patient treatment according to the current clinical practice: conventional chemotherapy for all patients regardless of *EGFR* mutation status.

### Model Structure and Methodology

**Fig 1:**
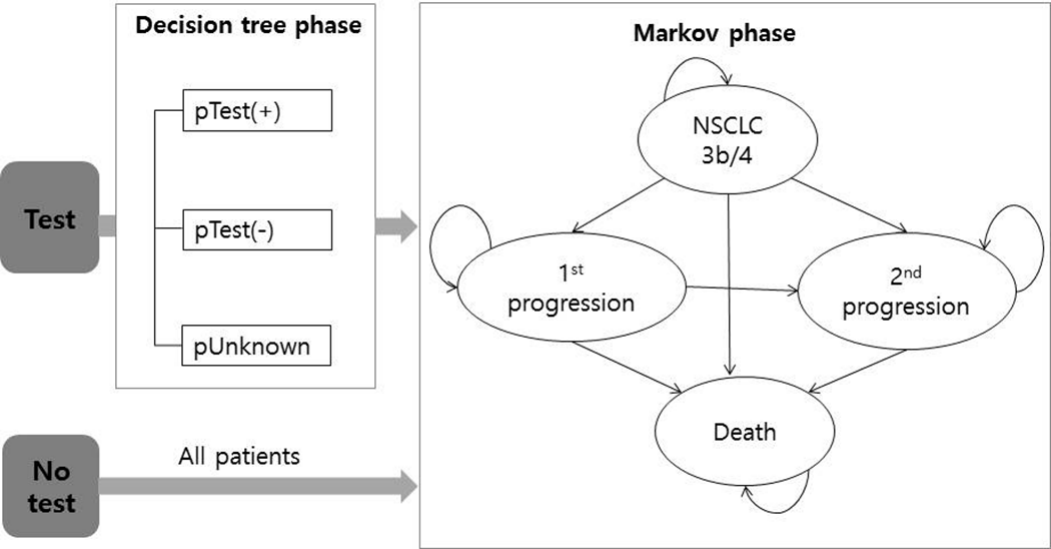
Model structure. NSCLC, non-small cell lung cancer; pTest+, proportion of test positives; pTest-, proportion of test negatives; pUnknown, proportion of unknown tests. Note: pTest+ = pTP + pFP [pTP = prevalence × sensitivity × (1-pUnknown), pFP = (1-prevalence) × (1-specificity) × (1-pUnknown)]; Test- = pTN + pFN [pTN = (1-prevalence) × specificity × (1-pUnknown), pFN = prevalence × (1-sensitivity) × (1-pUnknown)]. ※ pTP, pFP, pTN, and pFN is the proportion of true positive, false positive, true negative, and false positive results, respectively. Prevalence is the percentage of patients harboring *EGFR* mutations in stage 3b and 4 lung adenocarcinoma.

The model consisted of two components: a decision model and a Markov model. A decision tree model was used to select an appropriate treatment regimen according to test result in the testing strategy, and a Markov model was used to simulate NSCLC progression in both the testing and no-testing strategies. To calculate the proportions of each decision tree branch, we estimated the positive and negative predictive values of *EGFR* mutation testing based on test sensitivity and specificity, prevalence of *EGFR* mutations in patients with stage 3b and 4 lung adenocarcinoma, and the proportion of unknown test results which are the cases where EGFR mutation status cannot be determined due to inadequate tumor tissue. Unknown result may require rebiopsy. The Markov cycle length was one month, and the time horizon was five years (Fig 1). To simplify the model, it was assumed that cancer treatment was permitted up to the second progression, and best support care (BSC) was provided to control symptoms after the second progression.

The type of drug therapy was selected based on treatment guidelines [6–8], the medical advice of oncologists, and previous cost-effectiveness studies [5,12,14]. In the testing strategy, *EGFR* mutation-positive patients received first-line erlotinib treatment, second-line chemotherapy with cisplatin plus pemetrexed, and then BSC if all treatments failed. *EGFR* mutation–negative or test-unknown patients received first-line cisplatin plus pemetrexed treatment, second-line docetaxel, and then BSC. Meanwhile, patients in the no-testing strategy received first-line cisplatin plus pemetrexed treatment, second-line erlotinib, and then BSC. We assumed that all patients were administered four cycles of chemotherapy.

Progression-free survival (PFS), overall survival (OS), and adverse events were included as clinical outcomes. Disutilities for adverse events were applied to the first cycle in the Markov model for a single cycle. To apply time-variant Markov transition probabilities, we used data obtained from the Kaplan-Meier (K-M) curve, which was extracted using an image-digitizing program [16]. We extrapolated up to 60 months, applying frequently used methods: (1) to fit the curve and minimize the sum of squares between actual data obtained from the K-M curve and estimated data (least square method), (2) to regress ln(-ln(S(t)) vs. ln(t) (regression method). The mean of the two methods was used for the base case model.

The study was analyzed from a healthcare–payer perspective and included analysis of direct medical costs and excluded uninsured benefits. Costs were estimated using a macro-cost approach with the claims data of the 2011 Health Insurance Review and Assessment Services-National Patient Sample (HIRA-NPS). All the costs were adjusted to 2014 prices with the Consumer Price Index and were reported in US dollars (1 US dollar = Kor ₩1,012.1). The base case model applied a 3% discount rate.

The data for clinical outcomes and utilities were obtained from published studies. The result was reported as an incremental cost-effectiveness ratio (ICER), and sensitivity analyses were performed to explore uncertainties. Cost data were analyzed using SAS® 8.2 software, and the cost-effectiveness model was analyzed using Treeage® pro 2014 software.

### Epidemiologic Data

To investigate the prevalence of *EGFR* mutations in stage 3b and 4 lung adenocarcinoma in Korea, we performed a literature search using the Medline and Korean search engine (RISS). Inclusion was limited to studies published after 2005 with greater than 50 samples. Only one study by Choi et al. [17] was found to be valid. Choi et al. analyzed retrospective cohorts (n = 1,503) of all patients treated for stage 3b or 4 NSCLC between 2007 and 2010 in the Samsung Medical Center, one of the major hospitals in Korea. Adenocarcinoma composed 75% of the cohorts. The prevalence of *EGFR* mutations was 36.3% in stage 3b and 4 NSCLC and 39% in stage 3b and 4 adenocarcinoma (Table 1). The proportion with an unknown test result was 11%, which was decided based on expert opinions and was considered to be comparable to results from another study [11] where rebiopsy was not performed in 10% despite inadequate tumor tissue and ‘still unknown test result after rebiopsy‘ was 1.5%.

**Table 1:** Input parameters.

Item	Value	95% CI or SD	Source
Prevalence of *EGFR* mutations in adenocarcinoma	39%		[17]
Prevalence of *EGFR* mutations in stage 3b and 4 NSCLC	36.3%		[17]
Test sensitivity	98.4%		[18–22]
Test specificity	89.2%		[18–22]
Unknown %	11%		Assumption
Cost of *EGFR* testing (US $)	104		Medical fee
Total treatment cost per one cycle (US $)		(SD)	
During ERT administration (all cycles)	2,113	2,381	2011 Claims data
During CPEM administration	4,157	3,301	2011 Claims data
During CPEM non-administration	503	471	2011 Claims data
During CGEM administration	3,327	2,943	2011 Claims data
During CGEM non-administration	454	455	2011 Claims data
During DOX administration	3,300	2,378	2011 Claims data
During DOX non-administration	616	430	2011 Claims data
During PEM administration	4,501	2,708	2011 Claims data
During PEM non-administration	594	603	2011 Claims data
During BSC (all cycles)	1,038	884	[23]
Utilities		(95% CI)	
NSCLC	0.6532	0.6096/0.6968	[24]
Progression	0.4734	0.4309/0.5159	[24]
BSC	0.4734	0.4309/0.5159	[24]
Disutility by adverse side effect			
- Neutropenia	-0.0897	0.0595/0.1200	[24]
- Febrile neutropenia	-0.0900	0.0580/0.1220	[24]
- Fatigue	-0.0735	0.0373/0.1097	[24]
- Nausea & vomiting	-0.0480	0.0163/0.0797	[24]
- Diarrhea	-0.0468	0.0164/0.0772	[24]
- Hair loss	-0.0450	0.0160/0.0740	[24]
- Rash	-0.0325	0.0095/0.0555	[24]
- Anemia	-0.0730	0.0377/0.1083	[14]
Utility in PO.	+0.02	Not reported	[25]
Disutility by IV inj.	-0.043	0.0038/0.0822	[14]

(1 US dollar = Kor ₩1,012.1). 95% CI, 95% confidence interval; SD, standard deviation; *EGFR*, epidermal growth factor receptor; NSCLC, non-small cell lung cancer; ERT, erlotinib; DOX, docetaxel; CGEM, cisplatin plus gemcitabine; CPEM, cisplatin plus pemetrexed; PEM, pemetrexed; BSC, best supportive care; PFS, progression-free survival; OS, overall survival.

### Diagnostic Accuracy

With regard to the average diagnostic accuracy for representative *EGFR* mutation testing techniques, we used a systematic review study on diagnostic accuracy by Ellison et al. [18]. Since the study did not include the Therascreen® *EGFR* RGQ PCR kit or the Cobas® CDx as approved by the FDA, we performed an additional literature search for these two techniques. We used the results of sensitivity and specificity testing as evaluated by direct sequencing (Sanger sequencing) as a reference standard; the average sensitivity and specificity were 98.4% and 89.2%, respectively (Table 1).

### Clinical Outcomes

We adopted systematic approaches to literature searches from Medline to obtain the clinical data needed for the Markov branches. Separate searching strategies were developed for the intervention required in each Markov branch, based on first-line or second-line treatment, or mutation status. The search was limited to randomized controlled trials (RCT), published after 2005, and written in English or Korean; however, the type of control was not defined. The major inclusion criteria were studies reporting survival curves for outcomes, a target population of patients with stage 3b and 4 NSCLC, and an Eastern Cooperative Oncology Group (ECOG) performance status of 0–1 ≥ 80% of included patients. The weighted average was calculated by the sample size if more than one study was selected.

Studies meeting the inclusion criteria for most Markov branches are listed as follows: PFS with erlotinib as the first-line treatment in *EGFR* mutation-positive patients [15,19,26], PFS and OS with cisplatin plus pemetrexed as a second-line treatment in mutation-positive patients [27], PFS with cisplatin plus pemetrexed as a first-line treatment in mutation-negative patients [28], PFS and OS with docetaxel as a second-line treatment in mutation-negative patients [29], PFS and OS with cisplatin plus pemetrexed as a first-line treatment in the no-testing strategy [30,31], PFS and OS with cisplatin plus gemcitabine as a first-line treatment in the no-testing strategy [31–39], PFS and OS with erlotinib as a second-line treatment in the no-testing strategy [40–51], and OS with BSC [52]. The study for second-line cisplatin plus pemetrexed treatment in mutation-positive patients was a single-arm study. The OS of first-line erlotinib treatment in mutation-positive patients and first-line cisplatin plus pemetrexed treatment in mutation-negative patients were replaced with the OS of all patients in the no-testing strategy. According to the results, the PFS of erlotinib in *EGFR* mutation-positive patients produces a distinctively better result than other cases. The integrated results applied to the model are provided in a S1 and S2 Figs as form of survival curves.

### Costs

We considered only reimbursed direct medical costs, which included inpatient, outpatient, and pharmacy costs based on health insurance claims data. To assess the costs, we selected patients diagnosed with lung cancer (C34) using the Korean Standard Classification of Diseases version 6 (KCD-6) codes. To select patients with stage 3b and 4 NSCLC, we used an operational definition of all the cases of patients who received chemotherapy and excluded patients who had surgery. The main assumption in the cost estimation was that costs only depended on the treatment regimen, regardless of first-line or second-line treatment, and regardless of *EGFR* mutation status.

A total of 378 patients were analyzed for the cost assessment. According to the descriptive analysis, 224 of the patients were men (59.3%), the average age was 64 years, and the majority (64.2%) was in their seventies. Excluding outliers, the total average annual cost for NSCLC treatment was $17,785 USD and the average inpatient cost was $9,584 USD. Inputs of costs for Markov states are presented in Table 1.

Costs for BSC were obtained from a published study [23] that investigated terminal cancer patients under hospice care; the average medical cost for these patients was $1,038 USD per month (Table 1).

### Utilities

The two representative studies on the quality of life of NSCLC patients were performed by Nafees et al [24], and Doyle et al. [53]. We applied the results of the former to the model because that study was more relevant in terms of health statuses. The study determined the utilities of the health statuses of patients with stage 3b and 4 NSCLC and the disutilities of grade III-IV toxicities under situations where metastatic NSCLC patients received second-line chemotherapy. Data on disutility in anemia was taken from a study by Westwood et al. [14]. We assumed that the adverse events occurred during the first cycle, and that adverse event rates relied on the type of treatment regimen, regardless of whether it was first-line or second-line treatment. Finally, in case of an oral medication, an adjustment of 0.02, obtained from a study by Tabberer et al. [25], was added to the utilities investigated by Nafees et al., because the study was performed using intravenous treatments of pemetrexed and docetaxel.

### Sensitivity Analysis

Sensitivity analyses were conducted for uncertainties in different treatment effects and different utilities. Additionally, one-way sensitivity analyses were conducted for the test cost, analysis cycle, prevalence of *EGFR* mutations, proportion of unknown tests, test accuracy, and NSCLC treatment costs.

## Results

The base case analysis (Table 2) showed that the testing strategy would be the dominant option, with greater effectiveness and lower costs than the no-testing strategy: the average medical costs were $23,952 USD in the no-testing strategy and $23,334 USD in the testing strategy, and quality-adjusted life-years gained (QALYs) were 0.556 and 0.635 respectively. The sensitivity analyses generally demonstrated robust results. However, both the incremental cost and incremental QALY of the testing strategy increased in the model that substituted the OS with erlotinib in mutation-positive patients with the OS achieved with cisplatin plus pemetrexed treatment in all patients of the no-testing strategy, and in the CGEM-PEM model that applied first-line cisplatin plus gemcitabine and second-line pemetrexed for chemotherapy treatment branches. As a result, the testing strategy was not dominant, but still a cost-effective option. In particular, application of the prolonged OS of erlotinib resulted in a greater increase in the cost than the effect, meaning that erlotinib may not be effective enough to offset the increased cost.

**Table 2:** Results of cost-effectiveness in the base case and sensitivity analyses.

Strategy	Cost	Incr Cost	Efficacy	Incr Eff	ICER
	(US$)	(US$)	(QALY)	(QALY)	(US$/QALY)
Base case: adenocarcinoma 39%, discount rate 3%, sensitivity 98.4%, specificity 89.2%					
Test	23,334		0.63522		
No test	23,952	619	0.55621	-0.07901	dominated
Sensitivity analyses of different probabilities of PFS or OS					
1) Extrapolation: Regression ln(-ln(S(t)) vs. ln(t)					
Test	23,504		0.65240		
No test	24,048	544	0.57575	-0.07665	dominated
2) Extrapolation: Least square estimation					
Test	22,715		0.60107		
No test	23,747	1,032	0.53264	-0.06843	dominated
3) Substitution OS of erlotinib in M+ by OS of cisplatin plus pemetrexed in all (no-testing)					
No test	23,952		0.55621		
Test	24,064	111	0.66774	0.11153	999
Sensitivity analyses of different treatment regimens (health outcomes and costs)					
1) ERT-PEM model (1^st^ line ERT, 2^nd^ line PEM in M+)					
Test	23,300		0.63087		
No test	23,952	653	0.55621	-0.07466	dominated
2) CGEM-PEM model [1^st^ line CGEM, 2^nd^ line PEM in M- and 1^st^ line CGEM in all (no-testing)]					
No test	19,954		0.55591		
Test	21,528	1,574	0.64013	0.08421	18,687
Sensitivity analyses of different utilities					
1) Side-effect induced disutility during the whole period of drug administration					
Test	23,334		0.62775		
No test	23,952	619	0.54760	-0.08015	dominated
2) Disutility in intravenous medication according to Wang et al. [54]					
Test	23,334		0.61087		
No test	23,952	619	0.53137	-0.07950	dominated

(Exchange rate: 1 US$ = 1,021 KRW); ICER, Incremental cost-effectiveness ratio; OS, overall survival; M+, *EGFR* mutation positive; M-, *EGFR* mutation negative; ERT, erlotinib; PEM, pemetrexed; CPEM, cisplatin plus pemetrexed.

**Fig 2:**
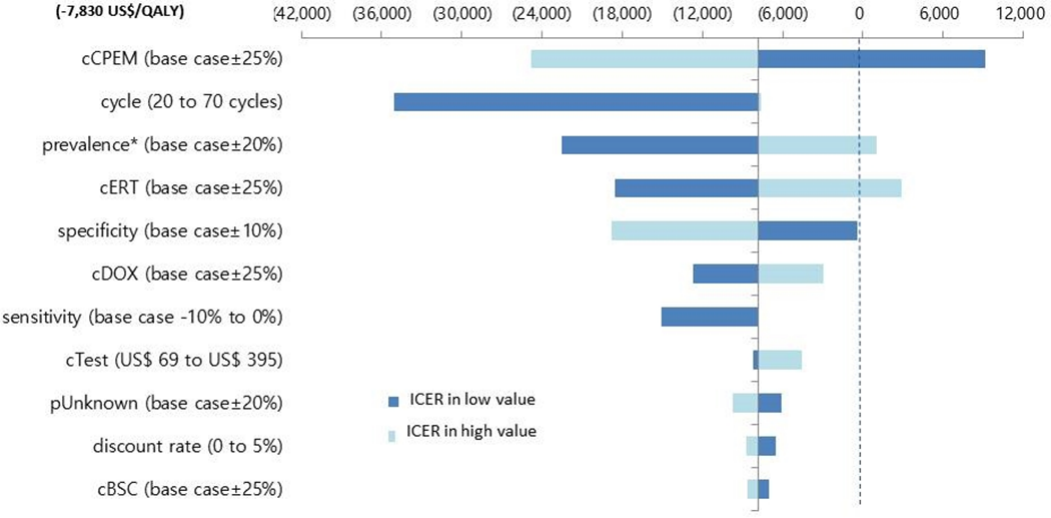
Tornado diagram of one-way sensitivity analysis. * Prevalence of *EGFR* mutation. cERT, medical cost of erlotinib; cCPEM, medical cost of cisplatin plus pemetrexed; cDOX, medical cost of docetaxel; cTest, cost of genetic test; cBSC, medical cost of best supportive care in terminal cancer patients; pUnknown, proportion of unknown tests.

The results of the one-way sensitivity analyses are presented as a tornado diagram in Fig 2. The most sensitive parameter was the medical cost of cisplatin plus pemetrexed therapy, but the testing strategy was still cost-effective within the lower and upper 25% of medical costs. The medical cost of BSC was the least sensitive of all costs. The ICERs increased with increasing cycles, prevalence of *EGFR* mutations, medical cost of erlotinib or docetaxel therapy, and test cost. A high prevalence of *EGFR* mutations incurred a greater increase in the cost than in the effect. This resulted in an increase in ICER. The results with regard to the proportion of unknown tests or sensitivity of a test also showed the same pattern, i.e., the ICER increased because of increased costs when the number of patients receiving first-line erlotinib treatment increased.

## Discussion

The study was conducted to investigate the cost-effectiveness of CDx and targeted therapy in patients with stage 3b and 4 lung adenocarcinoma. The base case and sensitivity analyses showed that treating patients as guided by *EGFR* mutation status was a dominant option. Nevertheless, without a cost-effective targeted therapy, the cost-effectiveness of CDx cannot be attained even if the technique is highly accurate; that is, the cost-effectiveness of CDx heavily depends on the cost and the effect of the targeted therapy in the context of the Korean healthcare system. From sensitivity analyses, we found that ICERs increased in situations where the number of patients receiving first-line erlotinib treatment increased, such as in the improved OS of first-line erlotinib treatment in mutation-positive patients, high prevalence of *EGFR* mutations, and low proportion of unknown test results. Those increased ICERs came from a much greater increase in the incremental cost than in the incremental effect. However, the benefit of first-line erlotinib treatment may be underestimated in this model, since the OS of first-line erlotinib treatment was substituted with the OS of cisplatin plus gemcitabine treatment in the no-testing strategy. The outcomes of the treatments tend to be better in *EGFR*-mutated patients than *EGFR* wild type or all patients, even with chemotherapy treatment [1].

Even though several studies have investigated the cost-effectiveness of *EGFR* mutation testing with accompanying targeted therapy (including gefitinib), any direct comparison of the results should be carefully made due to huge differences in the models and applied data among the studies, as well as differences among nations in terms of resource utilization, healthcare patterns, and medical costs. Nevertheless, by simply comparing the cost-effectiveness of first-line TKI therapy without considering other differences, our study showed more favorable results for the testing strategy than other studies [5,9–11]: the testing strategy was less costly and more effective than the no-testing strategy. All studies, including ours, demonstrated that the effect was improved in the testing strategy over the no-testing strategy. The incremental effects were 0.0307 QALYs in an Ontario study [5], 0.013 QALYs in a German study [11], 0.036 QALYs in a Japanese study [9], and 0.079 in our study. This greatest incremental effect in our study could be attributed to inclusion of the OPTIMAL trial [15] which reported the most favorable outcome for a TKI relative to other representative trials of TKIs such as the EURTAC trial [26] and the IPASS trial [1]. Varying prevalence of *EGFR* mutations in the studies may also account for the difference in incremental QALYs: 16.8% in the Ontario study, 12.8% in the study by Schremser et al., 32% in the study by Narita et al., and 39% in our study. However, the costs were more or less varied among the studies: decreased costs in the testing strategy in our study, and increased costs in the other studies. The difference in incremental costs may be due to variations in medical costs across countries and in conventional chemotherapy regimens across studies. The chemotherapy regimen applied in our model, cisplatin plus pemetrexed, was relatively costly compared to other models, such as cisplatin plus docetaxel in the Ontario model [5], carboplatin plus docetaxel and others in a German model [11], or carboplatin plus paclitaxel in a Japanese model [9]. The costly chemotherapy regimen would result in high costs for the no-testing strategy.

The prevalence of *EGFR* mutations in our study was obtained from a landmark study with large samples. Since all patients with stage 3b and 4 NSCLC were not tested for *EGFR* mutations in that study [17], the 39% *EGFR* prevalence reported in adenocarcinoma patients could be considered to be an overestimate. However, this prevalence did not differ greatly among other studies on the Asian population [9,55].

The present study is meaningful in some aspects. Differing from the previous studies [9–13] (except for the Ontario study), all the data available including the clinical outcomes of the intervention required in each Markov branch was obtained via a systematic review approach, instead of using one or two representative studies; as a result, the study provides up-to-date information on clinical efficacy with increased reliability and generalizability. In addition, we tried to reflect results seen in real-world settings by adopting a model of whole-disease progression from first-line treatment of stage 3b and 4 NSCLC to patient death.

However, the study has some limitations, mainly related to the clinical outcomes of the studies used. First, while we included RCT studies, the control effect was ignored because only a single arm from these studies was used, since the comparator was different among the studies available for each Markov branch. Second, no RCT study was found for second-line cisplatin plus pemetrexed treatment in mutation-positive patients, so that we used a single-arm study instead. Third, the study population was limited to patients with stage 3b and 4 lung adenocarcinoma, but most studies were conducted in all patients with stage 3b and 4 NSCLC. As a result, adenocarcinoma patients were analyzed as a subpopulation; thus, the population was not restricted to adenocarcinoma patients. However, these issues are likely to equally affect both strategies, and the cost-effectiveness may not change significantly. In addition, issues related to claims data could occur in the process of cost estimation, such as coding accuracy produced by the intention to maximize payment [56].

## Conclusion

From the Korean healthcare payer’s perspective using the Markov model of a one-month Markov cycle and five-year time horizon, the strategy of *EGFR* mutation testing before first-line erlotinib administration for advanced lung adenocarcinoma was the dominant option in the base case analysis, compared with the no-testing strategy. The incremental QALY was 0.07901, and the cost decrement of the testing strategy was $619 USD. The incremental QALY from our study was greater than other studies, in which the incremental QALYs ranged from 0.013 to 0.036. The sensitivity analyses were generally robust, but the ICERs increased in situations where the number of patients receiving erlotinib increased. Thus, we postulate that the cost-effectiveness of CDx depends on the cost-effectiveness of the targeted therapy. Further study is recommended to support the results of this study due to data limitations regarding clinical effects and costs.

This research was supported by the R&D Program for Society of the National Research Foundation (NRF) funded by the Ministry of Science, ICT & Future Planning (Grant number: 2013M3C8A1075908). Financial support from any other companies was not provided.

## Supporting Information

S1 Fig Survival curves of progression free survival (PFS) applied to the model. The survival cures were drawn by integrating results obtained from a systematic review. The number of included trials were three for first line erlotinib in M(+), one for second line cisplatin plus pemetrexed in M(+), one for first line cisplatin plus pemetrexed in M(-), one for second line docetaxel in M(-), two for first line cisplatin plus pemetrexed in all and twelve for second line erlotinib in all patients. M+, mutation positive; M-, mutation negative; ERT, erlotinib; CPEM, cisplatin plus pemetrexed; DOX, docetaxel.
(TIFF)

S2 Fig Survival curves of overall survival (OS) applied to the model. Due to lack of data, the OS of first line erlotinib in M(+) was replaced with the OS of first line cisplatin plus gemcitabine in all patients and first line cisplatin plus pemetrexed in M(-) was replaced with the OS of all patients. The number of included trials were nine for first line cisplatin plus gemcitabine in all, one for second line cisplatin plus pemetrexed in M(+), one for second line docetaxel in M(-), two for first line cisplatin plus pemetrexed in all and twelve for second line erlotinib in all patients. M+, mutation positive; M-, mutation negative; ERT, erlotinib; CPEM, cisplatin plus pemetrexed; DOX, docetaxel.
(TIFF)
