# Supplementary figures and images for: Economic Evaluation of Companion Diagnostic Testing for EGFR Mutations and First-Line Targeted Therapy in Advanced Non-Small Cell Lung Cancer Patients in South Korea
Source: PLoS One. 2016 Aug 2;11(8):e0160155. doi: 10.1371/journal.pone.0160155 (PMC4970739; doi:10.1371/journal.pone.0160155)

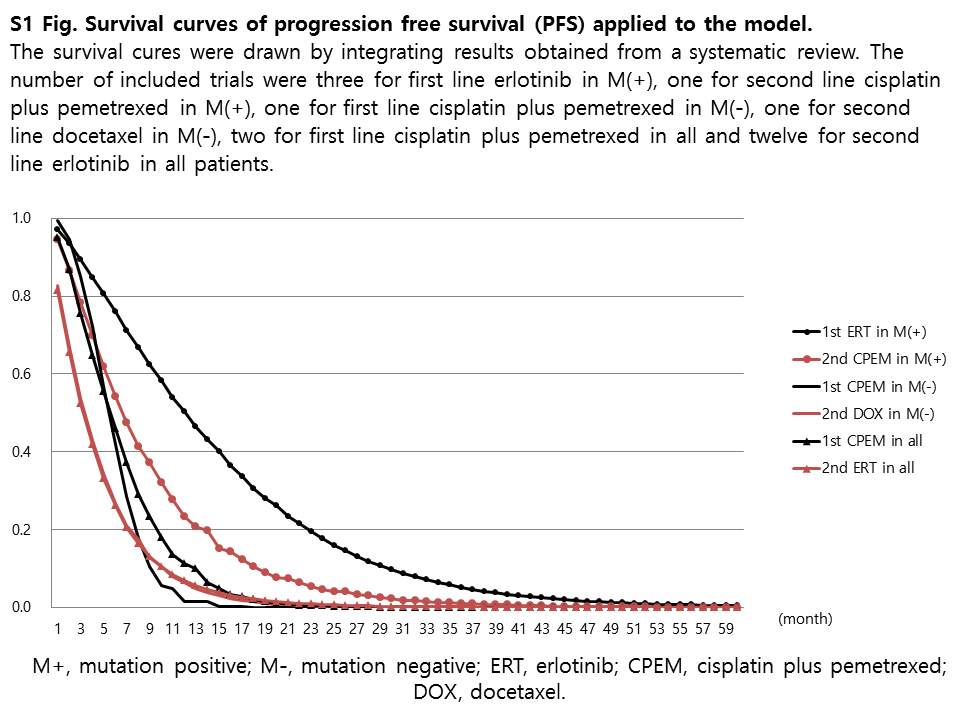

Supplement: S1 Fig — The survival cures were drawn by integrating results obtained from a systematic review. The number of included trials were three for first line erlotinib in M(+), one for second line cisplatin plus pemetrexed in M(+), one for first line cisplatin plus pemetrexed in M(-), one for second line docetaxel in M(-), two for first line cisplatin plus pemetrexed in all and twelve for second line erlotinib in all patients. M+, mutation positive; M-, mutation negative; ERT, erlotinib; CPEM, cisplatin plus pemetrexed; DOX, docetaxel. (TIFF) [file pone.0160155.s001.tiff]

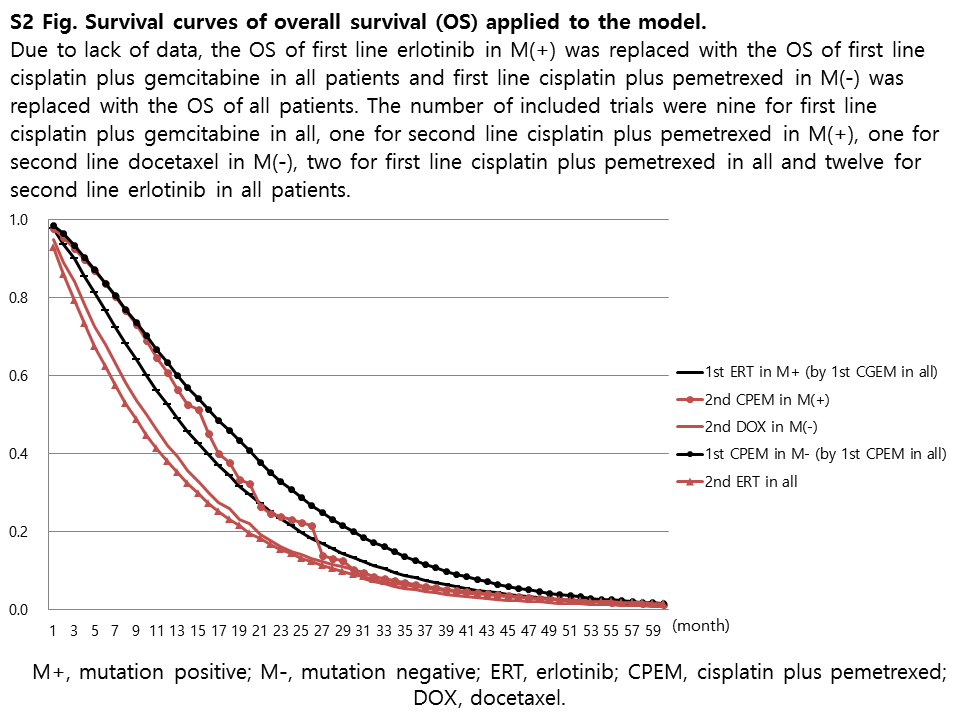

Supplement: S2 Fig — Due to lack of data, the OS of first line erlotinib in M(+) was replaced with the OS of first line cisplatin plus gemcitabine in all patients and first line cisplatin plus pemetrexed in M(-) was replaced with the OS of all patients. The number of included trials were nine for first line cisplatin plus gemcitabine in all, one for second line cisplatin plus pemetrexed in M(+), one for second line docetaxel in M(-), two for first line cisplatin plus pemetrexed in all and twelve for second line erlotinib in all patients. M+, mutation positive; M-, mutation negative; ERT, erlotinib; CPEM, cisplatin plus pemetrexed; DOX, docetaxel. (TIFF) [file pone.0160155.s002.tiff]
